# Sunitinib Does Not Accelerate Tumor Growth in Patients with Metastatic Renal Cell Carcinoma

**DOI:** 10.1016/j.celrep.2013.01.015

**Published:** 2013-02-07

**Authors:** Krastan B. Blagoev, Julia Wilkerson, Wilfred D. Stein, Robert J. Motzer, Susan E. Bates, A. Tito Fojo

**Affiliations:** 1 National Science Foundation, Arlington, VA 22030, USA; 2 Center for Cancer Research, National Cancer Institute, National Institutes of Health, Bethesda, MD 20892, USA; 3 Hebrew University, Jerusalem, Israel; 4 Memorial Sloan-Kettering Cancer Center, New York, NY 10065, USA

## Abstract

Preclinical studies have suggested that sunitinib accelerates metastases in animals, ascribing this to inhibition of the vascular endothelial growth factor receptor or the tumor’s adaptation. To address whether sunitinib accelerates tumors in humans, we analyzed data from the pivotal randomized phase III trial comparing sunitinib and interferon alfa in patients with metastatic renal cell carcinoma. The evidence clearly shows that sunitinib was not harm- ful, did not accelerate tumor growth, and did not shorten survival. Specifically, neither longer sunitinib treatment nor a greater effect of sunitinib on tumors reduced survival. Sunitinib did reduce the tumor’s growth rate while administered, thereby improving survival, without appearing to alter tumor biology after discontinuation. Concerns arising from animal models do not apply to patients receiving sunitinib and likely will not apply to similar agents.

## INTRODUCTION

Folkman first proposed angiogenesis, considered one of the hallmarks of cancer, as a therapeutic target more than four decades ago (Folkman, 1971; Folkman et al., 1971; Hanahan and Weinberg, 2011). In recent years the Food and Drug Administration has approved a number of drugs that in preclinical models target the molecular mechanisms thought to promote angiogenesis. In addition to targeting proteins involved in angiogenesis, most of these drugs have a broader spectrum and also influence other molecular processes. One of the targets of these “antiangiogenic” drugs is the family of vascular endothelial growth factors (VEGFs) and their receptors (VEGFRs), which promote new blood vessel growth during embryogenesis, wound healing, and tumorigenesis (Ferrara, 2009; Potente et al., 2011). VEGFs promote the dimerization of their targets (VEGFRs), membrane receptor tyrosine kinases (TRKs), leading to their phosphorylation. Subsequently, these phosphorylated receptors recruit intracellular proteins that upregulate pathways promoting blood vessel formation. In mammals, there are three receptors— VEGFR1, VEGFR2, and VEGFR3—and five ligand proteins—VEGFA, VEGFB, VEGFC, VEGF–D, and placenta growth factor. During the early stages of tumor growth, hypoxia upregulates VEGFA and VEGFR1 expression, making these proteins attractive as drug targets (Kowanetz and Ferrara, 2006).

Sunitinib is a tyrosine kinase inhibitor that has multiple targets (Papaetis and Syrigos, 2009; Heng and Kollmannsberger, 2010). It was approved for treatment of metastatic renal cell carcinoma (mRCC) on the basis of its ability to significantly delay the time to tumor progression in a randomized clinical trial and was subsequently shown to prolong survival (Motzer et al., 2007, 2009). A characteristic of renal cell carcinoma is its high vascularity, a property ascribed to alterations in the Von Hippel-Lindau tumor suppressor and HIF*α* that, in turn, mediate the overexpression of VEGF. Recently, however, studies in animal models have raised the possibility that antiangiogenic drugs might create a favorable environment for “acceleration” of metastasis (Ebos et al., 2009; Pà ez-Ribes et al., 2009). While an analysis of tumor growth kinetics in patients receiving the anti-VEGF antibody bevacizumab suggested that it could accelerate disease progression after the end of treatment (Stein et al., 2008), such evidence does not exist for small molecule inhibitors of the VEGFR (Stein et al., 2012). The study reported here assessed the possibility of acceleration of tumor growth in patients treated with the VEGFR inhibitor sunitinib. We analyzed data from the pivotal clinical trial comparing sunitinib and interferon alfa that led to sunitinib’s approval in metastatic renal cell carcinoma by the Food and Drug Administration in the United States and the European Medicines Agency in Europe (Motzer et al., 2007, 2009; Stein et al., 2012).

## RESULTS

**Figure 1.**
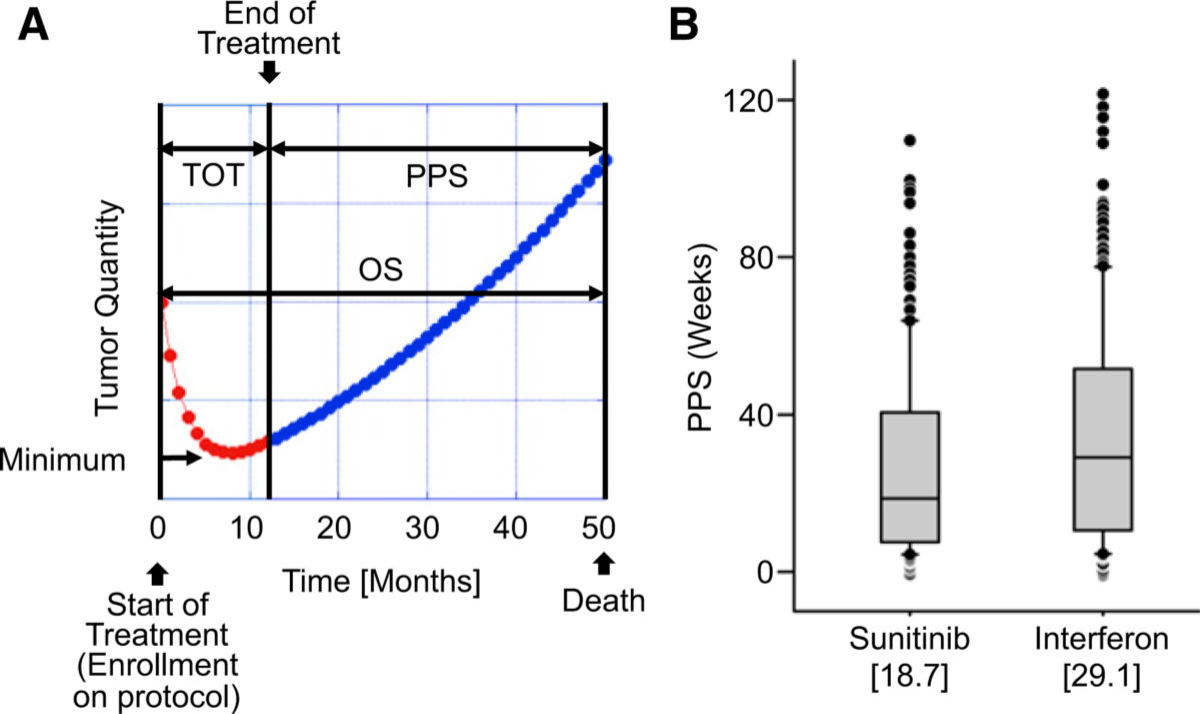
Definitions and Overall Results (A) Example of time course of tumor regression and growth in a “typical” patient receiving sunitinib and visual representation of terms used. The red symbols depict the actual tumor quantities measured while the patient was enrolled in the clinical trial. The blue symbols depict an estimation of tumor quantity after treatment was discontinued. Abbreviations are as follows: TOT, time on treatment; PPS, postprotocol survival; OS, overall survival. (B) PPS for patients randomized to receive sunitinib and those randomized to receive interferon alfa. The differences are statistically significant at p = 0.006.

Sunitinib treatment reduced the quantity of tumors in a majority of patients, and in approximately 30% of patients it reduced the tumor quantity to a level sufficient to qualify as a “response,” as defined by response evaluation criteria in solid tumors (RECIST) (Therasse et al., 2000). Figure 1A is a schematic representation of the time course of the measured tumor in a case where the tumor initially shrinks in size when treated with sunitinib. The figure presents the definitions used in this manuscript. Tumor shrinkage is observed initially because the fraction of tumor that is sensitive to the drug is greater, indeed often much greater, than the fraction that is resistant and the net quantity of tumor initially decreases over time. However, in >99% of patients, tumors eventually stopped shrinking as the resistant fraction became the predominant tumor fraction and tumor quantity then began to increase. This type of tumor-quantity kinetics can be described by a sum of two exponentials (Stein et al., 2008, 2012). The first exponential (d, decay or regression rate constant) describes the decrease in the quantity of the tumor fraction susceptible or sensitive to treatment. The second exponential (g, growth rate constant) describes the increase in the tumor quantity derived from cells resistant or insensitive to therapy. From the outset, both regression of sensitive tumor cells and expansion of the resistant cells occur simultaneously. Using this approach, one can discern a growth rate constant (g) even while the tumor is shrinking, and this growth rate constant explains the apparent deviation from a pure exponential decay of the tumor kinetics.

The postprotocol survival (PPS) endpoint, defined as the time interval from the time when protocol therapy ended to the time of death from any cause, was evaluated for both arms of the trial. As shown in Figure 1B, patients initially randomized to the interferon alfa arm had a longer median PPS in comparison to those assigned to receive sunitinib (medians: 29.1 versus 18.7 weeks, p = 0.006). While at first this might be interpreted as evidence that sunitinib “accelerated” the rate of tumor growth after its discontinuation, in fact, this most likely reflects the “postprotocol” treatment and management of patients (Motzer et al., 2007, 2009). Specifically, ≈60% patients initially randomized to interferon alfa eventually received sunitinib or another VEGFR inhibitor, agents that we now know delay progression when administered. By comparison, only 20% of patients initially randomized to sunitinib received a cytokine as subsequent therapy, meaning that a lower percentage of patients initially treated with sunitinib received as a postprotocol therapy a treatment known to be effective against metastatic renal cell carcinoma. Note here that at the time the study was conducted other therapies now available had not yet been approved for the treatment of metastatic renal cell carcinoma. We also cannot exclude the possibility that interferon alfa triggered an immune response in some patients that could have extended their lives “postprotocol.” The important point is that these confounding variables and not discontinuation of sunitinib could explain the differences in PPS. Furthermore, we would note here that patients randomized to receive sunitinib in fact survived longer than those assigned initially to interferon alfa, by 4.6 months (median overall survival [OS] 26.4 months for sunitinib versus 21.8 months for interferon alfa), again making any postprotocol acceleration highly unlikely (Motzer et al., 2009). Finally, we would note that patients initially randomized to sunitinib received the experimental therapy nearly 7 months longer than those initially assigned to interferon alfa (median durations of treatment 11 months versus 4 months) (Motzer et al., 2009).

**Figure 2.**
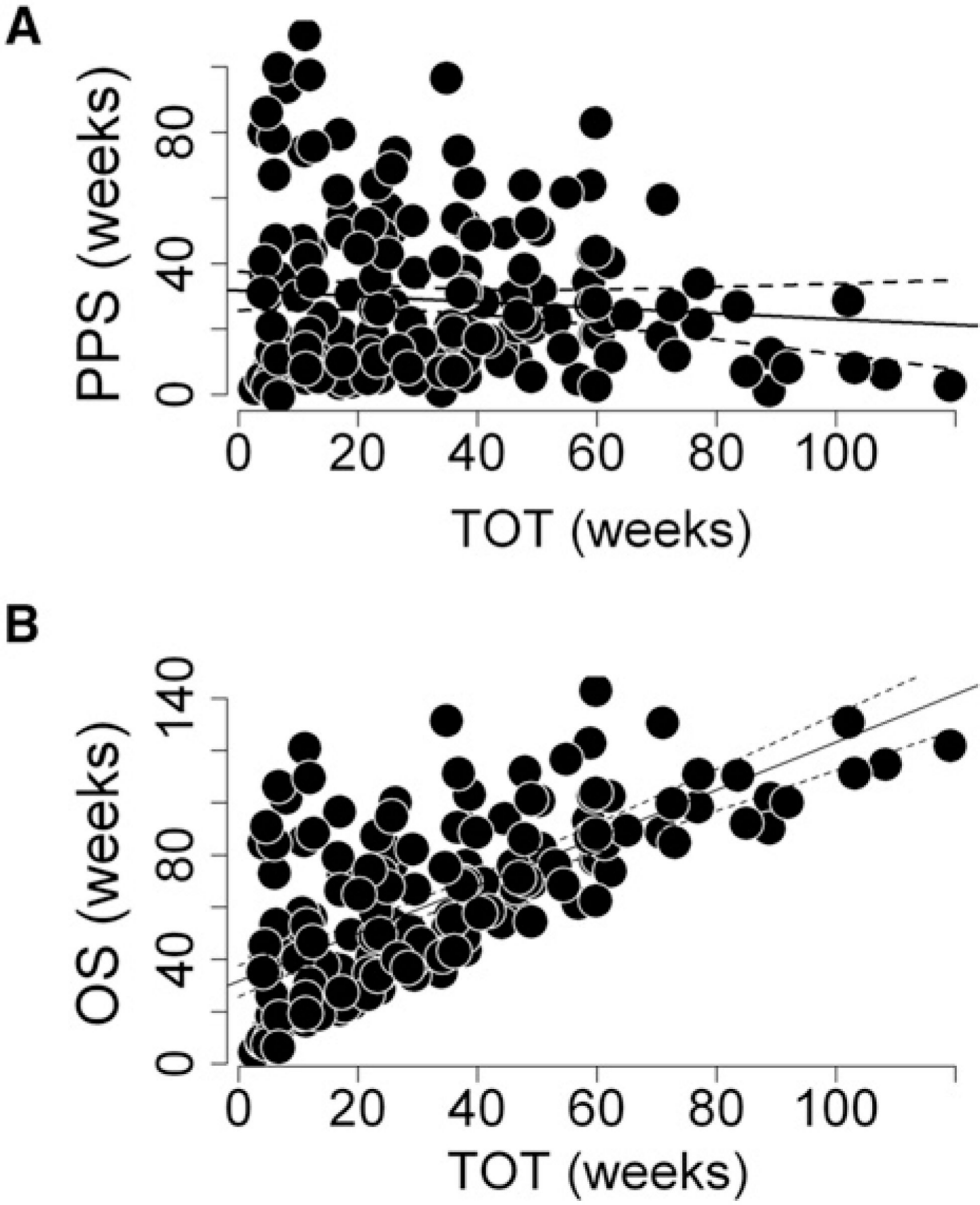
Effect of Sunitinib on Survival Outcomes (A and B) Correlations between TOT with sunitinib and either PPS (A) (Rsq = 0.003; p = 0.43) or OS (B) (Rsq = 0.49; p < 0.001), demonstrating the lack of an effect of duration of sunitinib exposure (TOT) on survival following the discontinuation of sunitinib (PPS).

While a comparison of patients according to their randomized treatment assignments cannot be done given the confounding variables discussed in the preceding paragraph, ample evidence within the sunitinib data argues strongly against the possibility that tumors “accelerated” after discontinuation of sunitinib therapy. For example, were treatment with sunitinib harmful, one would have expected greater harm to come to those who received sunitinib longer. However that was not the case, as shown when one examines the dependence of PPS on the time on treatment (TOT). TOT is the time from randomization to the time when treatment ceased for any reason, including death, toxicity, or an increase in tumor size by 20% in the longest diameter above the minimum length observed during the study. As shown in Figure 2, longer TOT, indicative of a patient receiving greater quantities of sunitinib, did not compromise a patient’s PPS (slope of regression line = −0.054, 95% confidence interval −0.189, 0.048), indicating that greater sunitinib exposure did not adversely impact PPS. TOT, however, did have an impact on OS, and, as Figure 2B shows, this was favorable, with patients receiving sunitinib for longer times also surviving longer. The latter is not surprising, given that longer treatment would have been administered to those benefiting from sunitinib and the longer the benefit the better the outcome.

**Figure 3.**
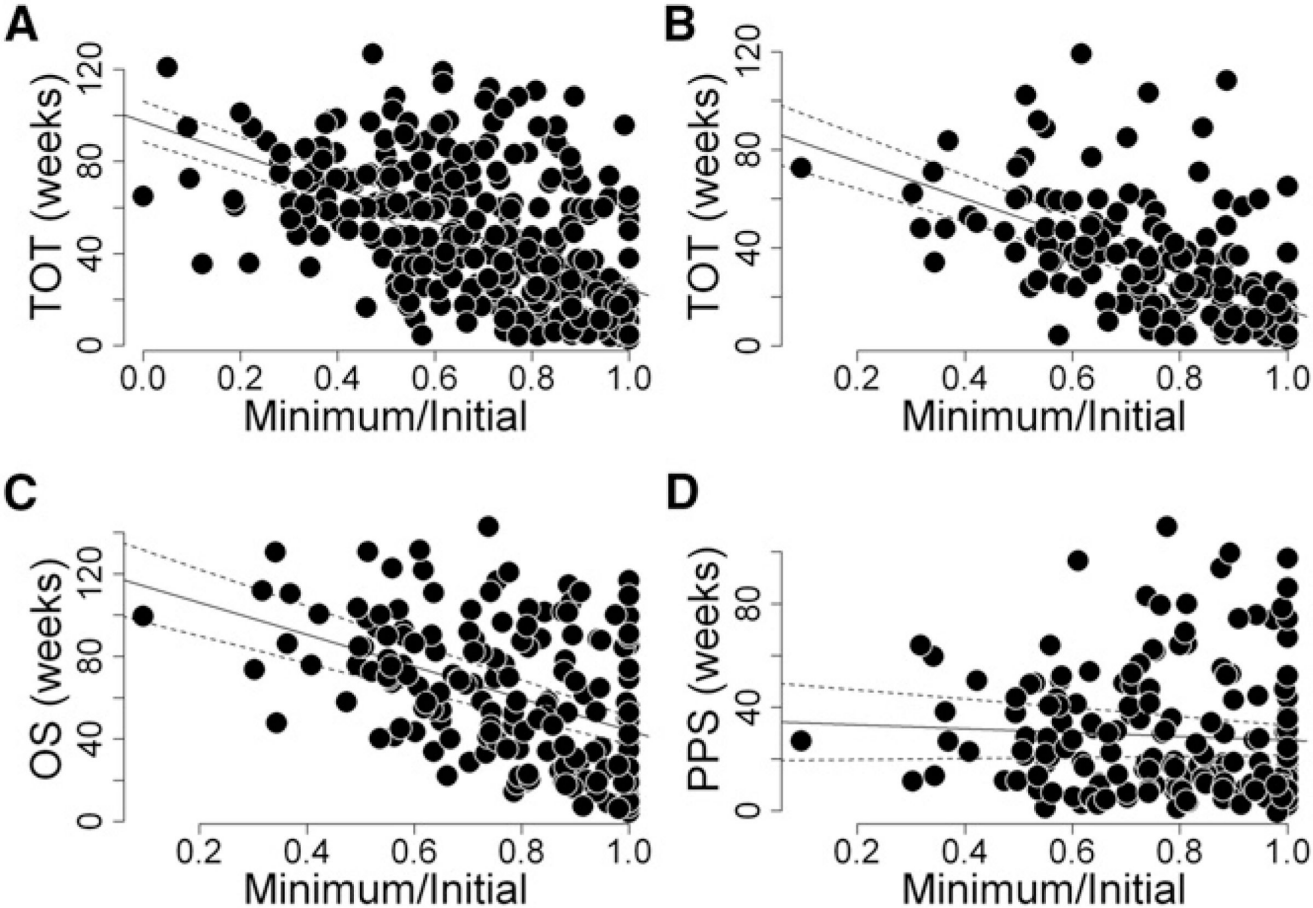
Tumor Response and Treatment Outcomes (A–D) Correlations between tumor response to sunitinib assessed as the ratio (Minimum/Initial) of the minimal tumor quantity (Minimum) measured while receiving therapy to the initial tumor quantity (Initial) at the start of therapy and either TOT (A, all patients, and B, all patients with date of death as in C and D), OS (C), or PPS (D). The data demonstrate the lack of an effect of sunitinib efficacy assessed as the ratio Minimum/Initial on PPS (R_sq_ = 0.0099, p = 0.1837), but modest effects on TOT (R_sq_ = 0.31, p < 0.001 for both A and B) and OS (R_sq_ = 0.21, p < 0.001).

Furthermore, if sunitinib were harmful, one could also expect that greater harm would come from a greater sunitinib effect on tumors, reflecting a greater effect on the tumor vasculature if indeed this is the drug’s target in humans. However, as shown in Figure 3, when examining the results obtained for TOT, PPS, and OS against the relative tumor quantity reduction, one cannot find any evidence of a harmful sunitinib effect. In this analysis, the sunitinib effect on the tumors of an individual patient—tumor response—is described by the extent of tumor shrinkage, defined as the ratio between the smallest tumor quantity ob- served during treatment (minimum) and the tumor quantity at the beginning of treatment (initial). Tumor response, analyzed as a continuous variable, correlates modestly with TOT (Figures 3A and 3B) and with OS (Figure 3C) but not with PPS (Figure 3D). Note here that these are relative values, not absolute ones; they reflect the drug’s effect on the tumor but do not consider the absolute quantity of tumor, so one should not expect those with the lowest minimal to initial tumor quantity ratios to necessarily survive longer, since both the quantity and the extent of shrinkage of that quantity are important for survival.

**Figure 4.**
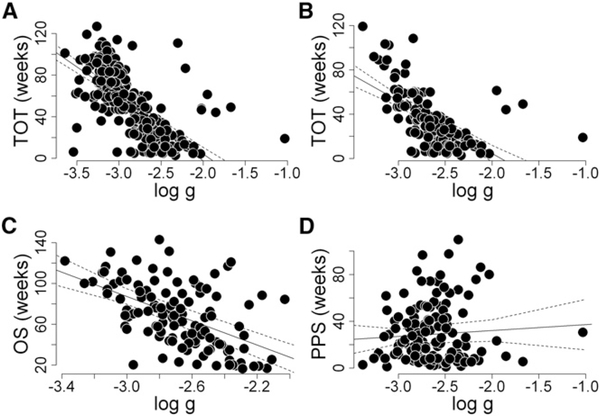
Tumor Growth Rates and Treatment Outcomes (A–D) Correlations between sunitinib efficacy in individual patients assessed as the on-study growth rate constant (g) derived using tumor measurements while receiving sunitinib therapy and either TOT (A, all patients; and B, all patients with date of death as in C and D), OS (C), or PPS (D). The data demonstrate the lack of an effect of sunitinib efficacy assessed as the on-study growth rate constant on PPS (R_sq_ = 0.000, p = 0.9586), but modest effects on TOT (R_sq_ = 0.41, p < 0.001 for A; and R_sq_ = 0.34, p < 0.001 for B) and OS (R_sq_ = 0.28, p < 0.001). Note that data similar to that depicted in (D) were previously published (Stein et al., 2012).

Similarly, if instead of using the minimal to initial tumor quantity as a measure of sunitinib effect on the tumors one uses the growth rate constant (g) calculated with the use of data obtained while patients received on-study treatment, once again one cannot find evidence that sunitinib was harmful. Specifically, one observes positive (favorable) correlations of g with TOT (Figures 4A and 4B), as well as with OS (Figure 4C), again demonstrating that a greater sunitinib effect on tumors, as evidenced by a slower g, is not harmful and does not reduce OS. Furthermore, the data show no correlation of on-study g with PPS (Figure 4D), again demonstrating that a greater sunitinib effect on the tumors did not compromise PPS. Thus, neither of two measures of a drug’s effect on the tumor—the tumor’s response, shown in Figure 3, nor the growth rate constant, depicted in Figure 4—support the notion that sunitinib was harmful; instead, they clearly demonstrate that a greater sunitinib effect was favorable.

## DISCUSSION

Using data from the pivotal trial comparing sunitinib versus inter- feron alfa in metastatic renal cell carcinoma, we asked whether sunitinib adversely impacts a patient’s clinical course—especially after discontinuation—perhaps by “accelerating” metastases, as has been suggested by preclinical studies (Ebos et al., 2009; Pàez-Ribes et al., 2009). No support for disease acceleration was found in patients receiving sunitinib. We demonstrate again that sunitinib therapy is beneficial for patients with mRCC. Specifically, we found no significant correlation between the length of time patients received sunitinib treatment and their postprotocol (postsunitinib) survival, indicating that longer exposure to sunitinib does not shorten survival after its discontinuation. Indeed, the longer patients stayed on treatment the longer their OS. Two other measures of sunitinib effect (the extent of tumor reduction and the tumor’s growth rate constant while the patient received sunitinib) also did not correlate with postprotocol (postsunitinib) survival, indicating that a greater sunitinib effect on the tumor does not portend a worse outcome after treatment is discontinued. Indeed, our analysis found no evidence to suggest that sunitinib alters tumor biology, other than slowing tumor growth while administered.

The difference between these results in humans and a previous study demonstrating that sunitinib discontinuation led to an “acceleration” of the disease may reflect the known limitations of otherwise valuable murine models (Ebos et al., 2009). A murine model in which a small, relatively “new” tumor is being assessed might differ from a situation in which a patient who has tumors that are more “established” and several centimeters in size. The former might be more dependent on “angiogenesis,” while the latter has an established vascular supply. We would note here that the mean and median tumor quantity “measured” in these patients was 14.3 and 11.2 cm, and, since in a majority of patients only a fraction of all tumor masses is measured, these patients clearly had tumors that were much larger in size than the tumors in the mice. Indeed, while sunitinib might have antiangio- genic effects in the small tumors found in mice, its activity in humans with metastatic renal cell carcinoma might primarily be antiangiogenic but might also be more complex. Given its broad inhibitory profile, inhibition of one or more kinases might also be important, although this has yet to be proven. An additional point is that mRCC presents as highly vascularized tumors, while the mouse studies used cancers that in humans are less vascular. In that respect, sunitinib may act differently on mRCC than on other cancer types. These possibilities require further study in humans.

We conclude thatthe clinical data for sunitinib do not indicate that the drug has any detrimental effect on established metastatic renal cell cancer. We would caution that we could not draw the same conclusion for smaller, microscopic tumors, such as those that might be encountered in an adjuvant setting. In an adjuvant setting, sunitinib is administered after a “complete” surgical resection in order to prevent or delay recurrence of occult or microscopic disease. Although there is nothing in the available evidence to suggest that adjuvant sunitinib will be harmful, in due course this question will be answered if patients enrolled in ongoing clinical trials evaluating adjuvant sunitinib experience “acceleration” of recurrences. Regarding the latter, we would note that now, more than 2 years after completing its target enrollment of 1,923 patients in September 2010, the ran- domized phase III ASSURE (Adjuvant Sorafenib or Sunitinib for Unfavorable Renal Carcinoma) trial has not reported an adverse outcome, making it less likely that an adverse acceleration will be eventually reported (http://www.cancer.gov/clinicaltrials/search/view?cdrid=478976&version=healthprofessional).

The available evidence unequivocally demonstrates that sunitinib reduces tumor growth while administered, improves OS, and does not appear to alter tumor biology after treatment discontinuation. While continuing treatment longer to avoid a return to “untreated” growth rates might be beneficial (Stein et al., 2012), we would state with confidence that sunitinib, and most likely similar drugs, can be given to patients with mRCCs without fear of accelerating tumor growth. Concerns arising from animal models do not appear to apply to mRCC patients receiving sunitinib.

## EXPERIMENTAL PROCEDURES

### Source of the Data and Definitions of Terms

Data, including tumor measurements, disease progression, and death dates, were anonymized and provided by Pfizer in spreadsheet format. The study was an international, multicenter phase 3 trial that enrolled 750 patients with previously untreated MRCC (Motzer et al., 2007, 2009). Patients were randomized to receive either interferon alfa at a dose of 9 million units subcutaneously three times each week or repeated 6-week cycles of sunitinib administered at a dose of 50 mg once daily for 4 weeks, followed by 2 weeks without treatment. The primary endpoint was progression-free survival (PFS). Secondary endpoints included the objective response rate (ORR), OS, patient-reported outcomes, and safety. PFS is defined as the duration of time from the start of treatment until (1) the time when the tumor quantity reaches a value 20% above the nadir in patients whose tumors shrank or (2) the time when tumor quantity reaches a value 20% above the initial in patients whose tumor quantity did not decrease, since in these patients the initial quantity is operationally their “nadir.” Additionally, in the rare patients who died before experiencing tumor “progression,” PFS was scored as the date of death. Assessments of tumor size at each interval provided the necessary measurements for calculation of individual tumor growth and regression rate constants. OS was defined as the time from randomization to death from any cause.

### Statistical Analysis and Estimation of the Growth Rate Constant

Linear regression via the SAS software was used to identify the presence or absence of statistically meaningful correlations. Chi-square tests and the p value were used as measures of statistical significance. The growth rate was estimated with the use of a previously described kinetic model (Stein et al., 2008, 2012).
